# Impaired Fat Absorption from Intestinal Tract in High-Fat Diet Fed Male Mice Deficient in Proglucagon-Derived Peptides

**DOI:** 10.3390/nu16142270

**Published:** 2024-07-14

**Authors:** Koki Nishida, Shinji Ueno, Yusuke Seino, Shihomi Hidaka, Naoya Murao, Yuki Asano, Haruki Fujisawa, Megumi Shibata, Takeshi Takayanagi, Kento Ohbayashi, Yusaku Iwasaki, Katsumi Iizuka, Shoei Okuda, Mamoru Tanaka, Tadashi Fujii, Takumi Tochio, Daisuke Yabe, Yuuichiro Yamada, Yoshihisa Sugimura, Yoshiki Hirooka, Yoshitaka Hayashi, Atsushi Suzuki

**Affiliations:** 1 Departments of Endocrinology, Diabetes and Metabolism, Fujita Health University School of Medicine, Toyoake 470-1192, Japan; koki.1990.11.9@gmail.com (K.N.); shinji0413jp@gmail.com (S.U.); sakai220@fujita-hu.ac.jp (S.H.); naoya.murao@fujita-hu.ac.jp (N.M.); 51019005@fujita-hu.ac.jp (Y.A.); hfuji@fujita-hu.ac.jp (H.F.); megumi03@fujita-hu.ac.jp (M.S.); haratake@fujita-hu.ac.jp (T.T.); sugiyosi@fujita-hu.ac.jp (Y.S.); aslapin@fujita-hu.ac.jp (A.S.); 2 Yutaka Seino Distinguished Center for Diabetes Research, Kansai Electric Power Medical Research Institute, Kyoto 604-8436, Japan; ydaisuke@kuhp.kyoto-u.ac.jp (D.Y.); yamada.yuuichiro@a3.kepco.co.jp (Y.Y.); 3 Laboratory of Animal Science, Graduate School of Life and Environmental Sciences, Kyoto Prefectural University, Kyoto 606-8522, Japan; s822731002@kpu.ac.jp (K.O.); ysk-iwasaki@kpu.ac.jp (Y.I.); 4 Department of Clinical Nutrition, Fujita Health University, Toyoake 470-1192, Japan; katsumi.iizuka@fujita-hu.ac.jp; 5 Graduate School of Bioscience and Biotechnology, College of Bioscience and Biotechnology, Chubu University, Kasugai 487-8501, Japan; gr23013-4634@sti.chubu.ac.jp (S.O.); m-tanaka@isc.chubu.ac.jp (M.T.); 6 Department of Gastroenterology and Hepatology, Fujita Health University, Toyoake 470-1192, Japan; fujiitd914@gmail.com (T.F.); takumitochiobiz@gmail.com (T.T.); yoshiki.hirooka@fujita-hu.ac.jp (Y.H.); 7 Department of Medical Research on Prebiotics and Probiotics, Fujita Health University, Toyoake 470-1101, Japan; 8 BIOSIS Lab. Co., Ltd., Toyoake 470-1192, Japan; 9 Center for One Medicine Innovative Translational Research, Gifu University Graduate School of Medicine, Gifu 501-1194, Japan; 10 Department of Diabetes, Endocrinology and Nutrition, Graduate School of Medicine, Kyoto University, Kyoto 606-8507, Japan; 11 Department of Endocrinology, Research Institute of Environmental Medicine, Nagoya University, Nagoya 464-8601, Japan; hayashiy@riem.nagoya-u.ac.jp; 12 Department of Endocrinology, Nagoya University Graduate School of Medicine, Nagoya 466-8550, Japan

**Keywords:** high-fat diet, glucagon, PPARα, CD36, lipid metabolism

## Abstract

(1) Background: Proglucagon-derived peptides (PDGPs) including glucagon (Gcg), GLP-1, and GLP-2 regulate lipid metabolism in the liver, adipocytes, and intestine. However, the mechanism by which PGDPs participate in alterations in lipid metabolism induced by high-fat diet (HFD) feeding has not been elucidated. (2) Methods: Mice deficient in PGDP (GCGKO) and control mice were fed HFD for 7 days and analyzed, and differences in lipid metabolism in the liver, adipose tissue, and duodenum were investigated. (3) Results: GCGKO mice under HFD showed lower expression levels of the genes involved in free fatty acid (FFA) oxidation such as *Hsl*, *Atgl*, *Cpt1a*, *Acox1* (*p* < 0.05), and *Pparα* (*p* = 0.05) mRNA in the liver than in control mice, and both FFA and triglycerides content in liver and adipose tissue weight were lower in the GCGKO mice. On the other hand, phosphorylation of hormone-sensitive lipase (HSL) in white adipose tissue did not differ between the two groups. GCGKO mice under HFD exhibited lower expression levels of *Pparα* and *Cd36* mRNA in the duodenum as well as increased fecal cholesterol contents compared to HFD-controls. (4) Conclusions: GCGKO mice fed HFD exhibit a lesser increase in hepatic FFA and triglyceride contents and adipose tissue weight, despite reduced β-oxidation in the liver, than in control mice. Thus, the absence of PGDP prevents dietary-induced fatty liver development due to decreased lipid uptake in the intestinal tract.

## 1. Introduction

Glucagon (Gcg), which is secreted from pancreatic α-cells, increases glucose production by promoting glycogenolysis and gluconeogenesis coupled with amino acid catabolism in the liver [1,2,3,4]. Gcg has been shown to participate in lipid metabolism by promoting lipolysis in adipocytes and fatty acid oxidation in the liver in in vitro experiments [5,6,7,8,9,10,11,12,13]. Various in vivo studies have been conducted to clarify whether enhancement or blockade of Gcg action has a beneficial effect on glucose metabolism and lipid metabolism [14,15,16,17,18,19,20]. Gcg receptor (GcgR) agonism has been shown to reduce body weight by increasing energy expenditure and/or suppressing food intake, thus improving insulin sensitivity, glucose tolerance, and decreased hepatic triglyceride contents in high-fat diet (HFD)-fed obese mice [15,16]. On the other hand, although the blockade of Gcg action improves insulin sensitivity and glucose metabolism, it increases the expression of lipogenic genes in the liver [20] and decreases hepatic triglyceride content in obese mice [16,17,18]. Thus, the role of glucagon in the beneficial effects on lipid metabolism is not clear.

Gcg, glucagon-like peptide-1 (GLP-1), and GLP-2 are produced from the same precursor, i.e., proglucagon. Plasma GLP-1 and GLP-2 levels are markedly increased in individuals with type 2 diabetes treated with a GcgR antagonist [21] as well as in GcgR-deficient mice [22]. In addition, GLP-1 not only potentiates glucose-induced insulin secretion as an incretin hormone, but also decreases body weight through suppression of feeding and gastric emptying [23]. Indeed, gastric emptying is reduced in GcgR-deficient (GcgRKO) mice [18,22] and food intake is reduced in ob/ob mice treated with a human monoclonal antibody that inhibits GCGR signaling [17]. GcgRKO mice fed HFD for 12 weeks show resistance to body weight gain and lower fat accumulation in the liver compared to control mice fed HFD for 12 weeks, in accordance with reduced food intake [18]. Thus, both changes in body weight and triglyceride accumulation in the liver in response to 12 weeks of HFD are altered in rodent models of Gcg deficiency, in which GLP-1 action is enhanced. Furthermore, it has been reported that GLP-1 reduces and GLP-2 increases postprandial lipid absorption from the intestinal tract by regulating the triglyceride-rich lipoprotein (TRL) apolipoprotein B48 (apoB48) levels [24,25,26], suggesting that both GLP-1 and GLP-2 participate in hepatic lipid accumulation. It therefore remains unclear whether the accumulation of triglycerides in the liver caused by the blockade of glucagon action is due to deficiency of glucagon action itself or to increased activity of GLP-1 and GLP-2. Thus, it is important to investigate hepatic triglyceride accumulation using an animal model exhibiting both defective glucagon and GLP-1 and GLP-2 action.

We previously reported that mice deficient in proglucagon-derived peptides (PGDPs), including Gcg, GLP-1, and GLP-2 (GCGKO), show resistance to obesity during 15 weeks of HFD feeding due to the increased expression levels of *Ucp1* (uncoupling protein 1), which is involved in heat production in white adipose tissue (WAT) and brown adipose tissue (BAT) [27]. However, the role of PGDPs in fat accumulation in the liver was not investigated in these studies. The present study was conducted to clarify the role of PGDPs in changes in lipid metabolism induced by short-term HFD feeding. As GCGKO mice display normal gastric emptying [19] and food intake on a standard diet [28], this model is considered suitable to analyze changes in lipid metabolism in the short term with minimal secondary effects on differential feeding and gastric emptying.

## 2. Materials and Methods

### 2.1. Experimental Mice and Diet

GCGKO mice with a C57BL/6 background were established and maintained [29]. GCGKO male mice and Gcg heterozygous female mice were intercrossed to obtain male GCGKO and Gcg heterozygous mice, which served as controls as previously described [19,30]. The mice used in the experiments were kept four per cage in identical cages after weaning under a standard 12 h light/dark cycle at room temperature 23 °C with free access to water and food.

The GCGKO and Gcg heterozygous mice were each divided into two groups: mice fed normal chow (NC) (CE-2 with carbohydrates at 58.5%, protein at 29.5%, and fat at 11.9% of total energy; CLEA Japan, Inc., Tokyo, Japan) and mice fed HFD (HFD32 with carbohydrates at 23.2%, protein at 20.1%, and fat at 56.7% of total energy; CLEA Japan, Inc., Tokyo, Japan) [27]. Male mice of 13 weeks of age were analyzed. All animal experimental procedures were carried out according to a protocol approved by the Institutional Animal Care and Use Committee of Fujita Health University.

### 2.2. Plasma Biochemical Analyses

Blood was collected from the tip of the tail and blood glucose levels were measured with an Antsense Duo Small Electrode Glucose Analyzer (Horiba, Kyoto, Japan). Blood samples were centrifuged (2000× *g*, 20 min, 4 °C) and the collected plasma samples were stored at −80 °C until analysis. Plasma hormone levels were measured using the following assays: insulin, Mouse/Rat Insulin ELISA Kit (Morinaga Institute of Biological Science, Kanagawa, Japan, Cat# M1108); FGF21 (Fibroblast growth factor 21), Mouse and Rat FGF21 ELISA Kit (BioVendor Inc., Brno, Czech Republic, Cat# RD291108200R); corticosterone, Corticosterone Enzyme Immunoassay Kit (ARBOR ASSAYS, Ann Arbor, MI, USA, Cat# K014-H1); FFA, NEFA C test (FUJIFILM Wako Pure Chemical Co., Osaka, Japan, Cat# 279-75401); triglyceride (Triglyceride E test, FUJIFILM Wako Pure Chemical Co., Osaka, Japan, Cat# 432-40201); and total cholesterol (LabAssay Cholesterol, Cholesterol Oxidase·DAOS method) (FUJIFILM Wako Pure Chemical Co., Osaka, Japan, Cat# LABCHO-M1).

### 2.3. Oral Fat Tolerance Test (OFTT) and Intraperitoneal Fat Tolerance Test (IPFTT)

The oral fat tolerance test (OFTT) [31] and intraperitoneal fat tolerance test (IPFTT) [32] were performed as previously reported. A total of 200 µL of olive oil (Sigma-Aldrich Co., LLC, Saint Louis, MO, USA, Cat# O1514) was administered by gavage to the mice after 5 h fasting, and blood was collected from the tail vein at 0, 1, 2, and 3 h during OFTT. Emulsified intralipid (intralipid −20%, emulsion, Sigma-Aldrich Co., LLC, Saint Louis, MO, USA, Cat# I141) was administered intraperitoneally at a dose of 5 mL/kg to mice starved for 16 h, and blood was collected from the tail vein at 0, 1, 2 and 4 h during IPFTT. Plasma triglyceride levels were measured using the Triglyceride E test (FUJIFILM Wako Pure Chemical Co., Osaka, Japan, Cat# 432-40201).

### 2.4. Isolation of RNA and Quantitative PCR Analysis of Various Tissues

Mice were sacrificed 1 week after the intervention of the diets. The livers, adipose tissues, and intestinal tracts were collected. The intestinal tract (duodenum) was sampled from its proximal end [28]. Extraction of RNA, synthesis of cDNA, and PCR (qPCR) were performed as previously reported [28,33]. Total RNA was reverse transcribed using the ReverTra Ace quantitative qPCR RT Master Mix (TOYOBO, Osaka, Japan, Cat# FSQ-201). After cDNA synthesis, qPCR was carried out in a 25 µL reaction containing THUNDERBIRD SYBR qPCR Mix (TOYOBO, Osaka, Japan, Cat# QPS-201). Quantitative real-time reverse transcription polymerase chain reaction was carried out using the ABI PRISM 7900HT Sequence Detection System (Applied Biosystems, Bedford, MA, USA). The primer sequences are shown in Table S1. The mRNA levels of the genes of interest were normalized by those of β-actin and expressed relative to that of control mice fed with NC.

### 2.5. Measurement of Food Consumption

Each experimental mouse was housed in an individual cage for 3 days for measurement of food consumption. Thereafter, food consumption over 24 h was measured as previously reported [28].

### 2.6. Measurement of Hepatic Triglyceride, FFA, Total Cholesterol and Glycogen Content

To determine the liver contents of FFA, triglyceride, and total cholesterol, 50 mg of each liver sample was homogenized with 500 µL (the liver triglyceride content) or 250 µL (the liver FFA content and the liver total cholesterol content) isopropanol. After centrifugation (2000× *g*, 15 min), the supernatant was analyzed using the NEFA C test (FUJIFILM Wako Pure Chemical Co.), Triglyceride E test (FUJIFILM Wako Pure Chemical Co.) [28], and LabAssay Cholesterol (Cholesterol Oxidase·DAOS method) (FUJIFILM Wako Pure Chemical Co.) [30].

To determine the liver glycogen level, 10 mg of each liver sample was homogenized with 200 µL ddH2O on ice and then boiled for 10 min to inactivate the enzyme. After centrifugation (18,000 rpm, 10 min), the supernatant was analyzed using a Glycogen Colorimetric Assay Kit II (Abcam, Tokyo, Japan, Cat# ab169558).

### 2.7. Immunoblotting Analysis

Immunoblotting analysis was carried out as previously reported [30] using HSL Antibody (1:1,000; Cell Signaling Technology, Waltham, MA, USA, Cat# 4107), phospho-HSL (Ser660) Antibody (1:1,000; Cell Signaling Technology, Waltham, MA, USA, Cat# 45804), ATGL Antibody (1:1,000; Cell Signaling Technology, Waltham, MA, USA, Cat# 2138), CD36 Antibody (1:1,000; Cell Signaling Technology, Waltham, MA, USA, Cat# 74002), or glyceraldehyde 3-phosphate dehydrogenase (GAPDH) antibody (1:4000; Cell Signaling Technology, Waltham, MA, USA, Cat# 2118). Western blotting analysis was performed using Amersham Image Quant 800 (IQ800) (Cytiva, Tokyo, Japan) in autoexposure mode. The contrast was automatically adjusted by the software. All samples were derived from the same experiment or parallel experiments and gels/blots were processed in parallel. Quantification was carried out with ImageJ software ver.1.53e (National Institutes of Health, Bethesda, MD, USA). HSL phosphorylation is expressed as the ratio of phospho-HSL (pHSL) relative to HSL. The expression levels of HSL, ATGL, and CD36 are normalized by the expression levels of GAPDH. The values in GCGKO mice are shown relative to control mice.

### 2.8. Histology

Tissue staining was performed according to previous reports. In brief, small intestine and colon samples were fixed with 4% paraformaldehyde phosphate buffer solution (FUJIFILM Wako Pure Chemical Co., Osaka, Japan, Cat# 161-20141) for 7 days and then embedded in paraffin. Sections (4 μm) were cut and stained with hematoxylin and eosin (HE) using a Hematoxylin and Eosin Stain Kit (SkyTec Laboratories, Inc., Logan, UT, USA, Cat# HAE-1) for histological assessment and with periodic acid-Schiff (PAS)/Alcian blue (AB) using Alcian Blue-PAS Stain Kit (SkyTec Laboratories, Inc., Logan, UT, USA, Cat# APS-1) for goblet cell enumeration. Stained tissue sections were viewed and photographed with BZ-II Analyzer software (BZ-II Analyzer software Ver. 2.2, Keyence, Osaka, Japan) using a BIOREVO BZ-9000 microscope (Keyence).

### 2.9. Measurement of Fecal Triglyceride, FFA, and Total Cholesterol

To determine fecal triglyceride content, 100 mg of dried feces (70 °C, 1 h) was homogenized using a bead crusher with 500 µL saline. Next, samples were mixed with chloroform and methanol (2:1) 1000 µL and shaken at 60 °C for 30 min. After centrifugation (1000× *g*, 10 min 25 °C), the chloroform layer was collected and samples were dried under vacuum. Samples were then dissolved in 100 µL isopropanol and analyzed using the following assays: FFA (NEFA C test, FUJIFILM Wako Pure Chemical Co.), triglyceride (Triglyceride E test, FUJIFILM Wako Pure Chemical Co.), and total cholesterol (LabAssay Cholesterol, Cholesterol Oxidase·DAOS method) (FUJIFILM Wako Pure Chemical Co.).

### 2.10. DNA Samples

Fecal samples from HFD-fed GCGKO mice and HFD-fed control mice were collected and stored at −80 °C. DNA was extracted from the fecal samples using a QIAamp DNA Stool Mini Kit (QIAGEN, Hilden, Germany, Cat# 51604) according to the manufacturer’s instructions.

### 2.11. 16S rRNA Next-Generation Sequencing

A primer set comprising Pro341F and Pro805R targeting the V3–V4 region of the bacterial 16S ribosomal RNA gene was used for PCR [34]. 16S rRNA next-generation sequencing (NGS) was conducted at Bioengineering Lab Co., Ltd. (Sagamihara, Japan). Paired-end sequencing (2 × 300 bp) was performed using the MiSeq platform (Illumina, San Diego, CA, USA) with the MiSeq Reagent Kit ver. 3 (Illumina). The data generated by the MiSeq sequencing system were processed, statistically analyzed, and visualized using the EzBioCloud 16S database and the 16S microbiome pipeline provided by ChunLab Inc. (Seoul, Korea EzBioCloud 16S-based MTP app, available at https://www.EZbiocloud.net accessed on 23 April 2024). Chao1 estimation, the Shannon diversity index, and the phylogenetic diversity index were utilized to assess alpha diversity in the microbial samples. Beta diversity, which reflects the overall phylogenetic distances among groups, was estimated using the Jenson–Shannon divergence and visualized through principal coordinate analysis (PCoA). Differences in alpha and beta diversity between groups were tested using the Wilcoxon rank-sum test and permutational multivariate analysis of variance (PERMANOVA) with 9999 permutations, respectively. Potential taxonomic biomarkers distinguishing each group were identified using the linear discriminant analysis effect size (LEfSe) algorithm following the default parameters.

### 2.12. Quantitative PCR Analysis of Gut Microbiomes

Quantitative PCR (qPCR) analysis was performed using QuantStudio 3 (Thermo Fisher Scientific, Waltham, MA, USA). The reaction mixture was prepared for each template DNA using the PowerTrack SYBR Green Master Mix (Thermo Fisher Scientific, Waltham, MA, USA, Cat# A46012) according to the manufacturer’s instructions. A primer set used to amplify Glycoside Hydrolase family 32 (GH32) genes of *P. distasonis* was *Parab.distasonis*_sig.GH32_F and *Parab.distasonis*_sig.GH32_R, as previously described [35]. For *Akkermansia muciniphila*, a primer set was designed to target the aspartic protease Amuc_1434* protein. Using BLASTp (version 2.15.0) on the National Center for Biotechnology Information (NCBI) website (https://www.ncbi.nlm.nih.gov/, accessed on 28 September 2022) we identified five genes encoding this protein in *Akkermansia muciniphila* strains. The designed primer set targets the consensus region of these genes, as detailed in Table 1. This set includes the forward primer Amuc_1434_139F (5′-CATYGGCTGTTATCCGCAGC-3′) and the reverse primer Amuc_1434_139R (5′-CTTCAACCCATCGCTCACCT-3′), targeting regions at positions approximately 570–589 bp and 689–708 bp, respectively, of the Amuc_1434* gene from the *A. muciniphila* strain DSM 22959 (GenBank: CP042830.1). The amplification cycle protocol included an initial denaturation at 95 °C for 2 min, followed by 40 cycles of denaturation at 95 °C for 10 s, annealing at 60 °C for 15 s, and extension at 72 °C for 15 s for *P. distasonis* or 20 s for *A. muciniphila.* A final extension was performed at 72 °C for 1 min. Primers *F_Bact* 1369 and R_*Prok* 1492 were used to amplify the total 16S rRNA genes, as previously described [36]. qPCR was performed similarly for *P. distasonis*. The levels of the *P. distasonis* GH32 gene and *A. muciniphila* Amuc_1434* gene in the DNA solution were quantified as the number of each gene relative to the total number of 16S rRNA genes.

### 2.13. Statistical Analysis

All results are expressed as mean ± SEM. Statistical analysis was performed using unpaired Student’s *t*-test or one-way ANOVA, followed by multiple comparisons corrected using Tukey’s method. A P value of less than 0.05 between groups was considered significant. GraphPad Prism 10 for Windows (GraphPad Software, San Diego, CA, USA) was used.

## 3. Results

### 3.1. The Effects of HFD Feeding on the Weight of Various Tissues and Hormones in the Presence and Absence of Proglucagon-Derived Peptides (PGDPs)

**Figure 1:**
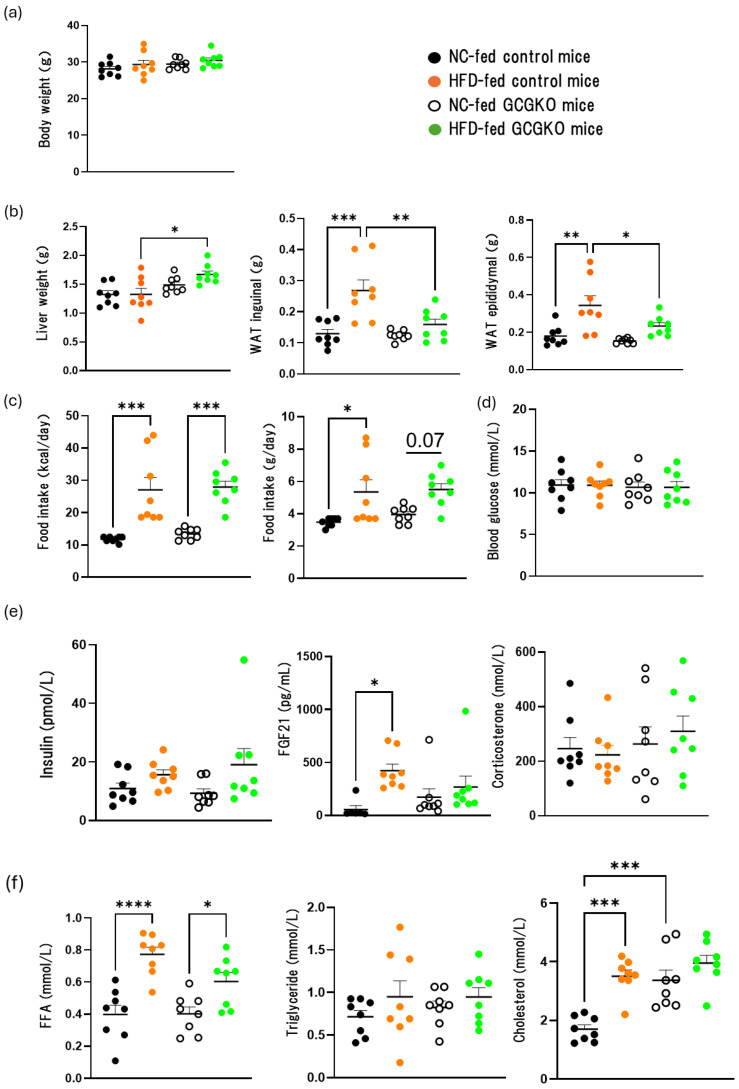
Effects of HFD feeding on the weight of various tissues and hormones in the presence and absence of proglucagon-derived peptides (PGDPs). (**a**) Body weight, (**b**) liver weight, inguinal and epididymal white adipose tissue (WAT) weight, (**c**) food intake (kcal/day, g/day), (**d**) blood glucose, plasma concentrations of (**e**) insulin, FGF21, corticosterone, and (**f**) FFA, triglyceride, cholesterol levels in normal chow (NC)-fed control mice (black dots; *n* = 8), high-fat diet (HFD)-fed control mice (orange dots; *n* = 8), NC-fed GCGKO mice (white dots; *n* = 8), and HFD-fed GCGKO mice (green dots; *n* = 8) after 1 week of intervention. (* *p* < 0.05, ** *p* < 0.01, *** *p* < 0.001, **** *p* < 0.0001). Data are expressed as mean ± SEM. Statistical comparisons were performed using one-way ANOVA with Tukey’s multiple comparisons test.

No significant difference in body weight was observed among the four groups (NC-fed control, HFD-fed control, NC-fed GCGKO, and HFD-fed GCGKO mice) after 7 days of differential feeding (Figure 1a). Liver weight was significantly increased in HFD-fed GCGKO mice compared to that in HFD-fed control mice. On the other hand, the weights of inguinal and epididymal WAT were significantly increased in HFD-fed control mice but not in HFD-fed GCGKO mice (Figure 1b). Food intake at the caloric base was similarly increased significantly by HFD-feeding in control and GCGKO mice (Figure 1c). These changes were accompanied by a significant increase in plasma FFA levels in both control and GCGKO mice (Figure 1f). Plasma cholesterol levels were higher in GCGKO mice than in control mice under NC, as previously described [20]. A significant increase due to HFD was observed in the control mice but not in GCGKO mice. No significant differences in blood glucose (Figure 1d), insulin, corticosterone (Figure 1e), or triglyceride (Figure 1f) levels were observed among the four groups. HFD feeding significantly increased plasma FGF 21 levels in control mice but not in GCGKO mice (Figure 1e).

### 3.2. Fat Accumulation in the Liver Was Decreased in HFD-Fed GCGKO Mice Compared to That in HFD-Fed Control Mice

**Figure 2:**
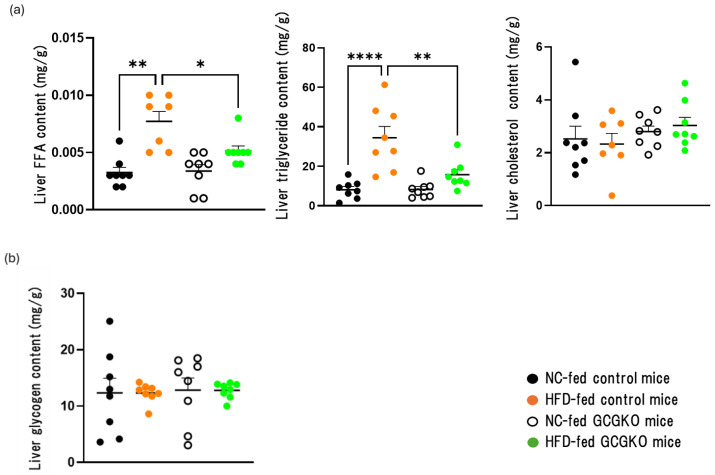
Fat accumulation in the liver of HFD-fed GCGKO mice and HFD-fed control mice. (**a**) FFA, triglyceride, and cholesterol content, and (**b**) glycogen content in NC-fed control mice (black dots; *n* = 8), HFD-fed control mice (orange dots; *n* = 8), NC-fed GCGKO mice (white dots; *n* = 8), and HFD-fed GCGKO mice (green dots; *n* = 8) in the liver after 1 week of intervention. (* *p* < 0.05, ** *p* < 0.01, **** *p* < 0.0001). Data are expressed as mean ± SEM. Statistical comparisons were performed using one-way ANOVA with Tukey’s multiple comparisons test.

Liver FFA and triglyceride contents were increased in HFD-fed control mice but such changes were attenuated in HFD-fed GCGKO mice (Figure 2a). Although uptake and/or synthesis of FFA and triglyceride is likely less in the absence of PGDPs, liver weight in GCGKO mice was increased compared to that in control mice. Liver cholesterol contents and liver glycogen contents did not differ among the four groups (Figure 2a,b), leaving the mechanism involved in increased liver weight in the GCGKO mice obscure.

### 3.3. Expression Levels of Genes Involved in β-Oxidation in the Liver of HFD-Fed GCGKO Mice and HFD-Fed Control Mice

**Figure 3:**
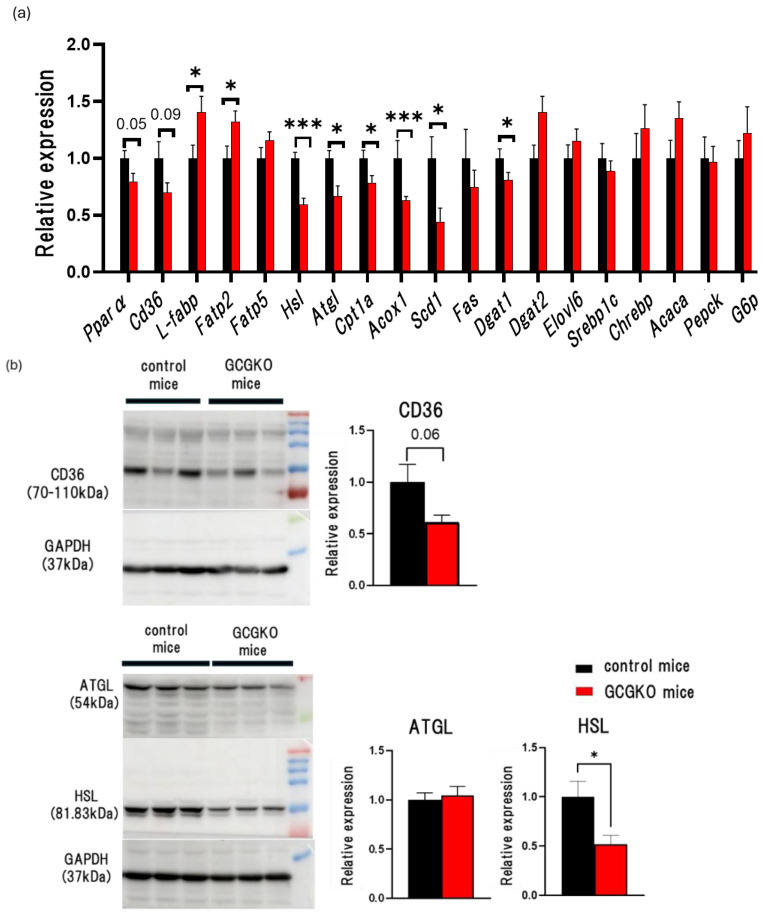
Gene expression levels related to β-oxidation in the liver of HFD-fed GCGKO mice and HFD-fed control mice. (**a**) mRNA expression levels of genes (*Pparα*, *Cd36*, *L-fabp*, *Fatp2*, *Fatp5*, *Hsl*, *Atgl*, *Cpt1a*, *Acox1*, *Scd1*, *Fas*, *Dgat1*, *Dgat2*, *Elovl6*, *Srebp1c*, *Chrebp*, *Acaca*, *Pepck*, and *G6p*) in the liver of HFD-fed control mice (black bars; *n* = 8) and HFD-fed GCGKO mice (red bars; *n* = 8). (**b**) Quantification of liver CD36, ATGL, and HSL in HFD-fed control and HFD-fed GCGKO mice. HFD-fed control mice (black bars; *n* = 6) and HFD-fed GCGKO mice (red bars; *n* = 6) in the liver after 1 week of intervention. (* *p* < 0.05, *** *p* < 0.001). Data are expressed as mean ± SEM. Statistical comparisons were performed using an unpaired Student’s *t*-test.

Glucagon promotes β-oxidation in the liver [5]. To determine whether changes in FFA uptake, lipolysis, and lipogenesis in the liver underlie reduced hepatic triglyceride contents in HFD-fed GCGKO mice, we analyzed gene expression levels and protein levels in the liver of HFD-fed mice. Among the genes involved in the transport of fatty acids, the expression levels of *Fatp2* (Fatty acid transport protein 2) and *L-fabp* (L-type fatty acid binding protein) were significantly higher in HFD-fed GCGKO mice than in HFD-fed control mice. On the other hand, the expression levels of *Cd36* (Cluster of differentiation 36), a scavenger receptor involved in the incorporation of the fatty acid transporter, were not increased but decreased in HFD-fed GCGKO mice, although the difference did not reach statistical significance. Among the genes involved in lipolysis and fatty acid oxidation, the expression levels of *Hsl* (Hormone Sensitive Lipase), *Atgl* (Adipose triglyceride lipase), *Cpt1a* (carnitine acyl transferase 1a), and *Acox1* (acyl-CoA oxidase 1) were significantly decreased in HFD-fed GCGKO mice compared to those in HFD-fed control mice, suggesting that β-oxidation is reduced in the liver of HFD-fed GCGKO mice. The expression levels of *Pparα* (peroxisome proliferator receptor alpha), the master regulator of hepatic lipid metabolism during fasting, tended to be lower in HFD-fed GCGKO mice. The expression levels of genes involved in lipogenesis and gluconeogenesis did not differ between the two groups, except for *Scd1* (stearoyl-Coenzyme A desaturase 1) (Figure 3a). Western blotting analyses showed that HSL expression levels in the liver were significantly decreased in HFD-fed GCGKO mice compared to those in HFD-fed control mice (Figure 3b). In parallel with the gene expression data, CD36 expression levels were statistically but marginally decreased in the HFD-fed GCGKO mice (Figure 3b).

These results suggest that reduced triglyceride accumulation in the HFD-fed GCGKO liver is unlikely to be due to increased fatty acid oxidization in the liver.

### 3.4. The Effects of HFD Feeding and Proglucagon-Derived Peptides (PGDPs) on Adipose Tissue

**Figure 4:**
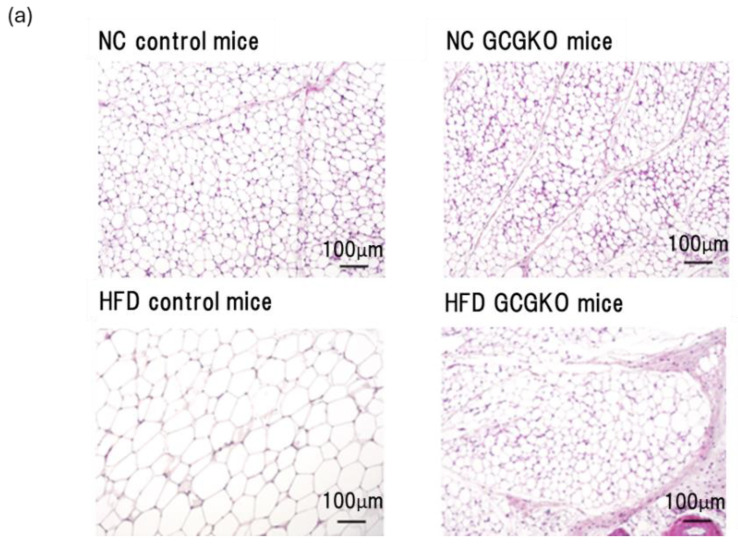

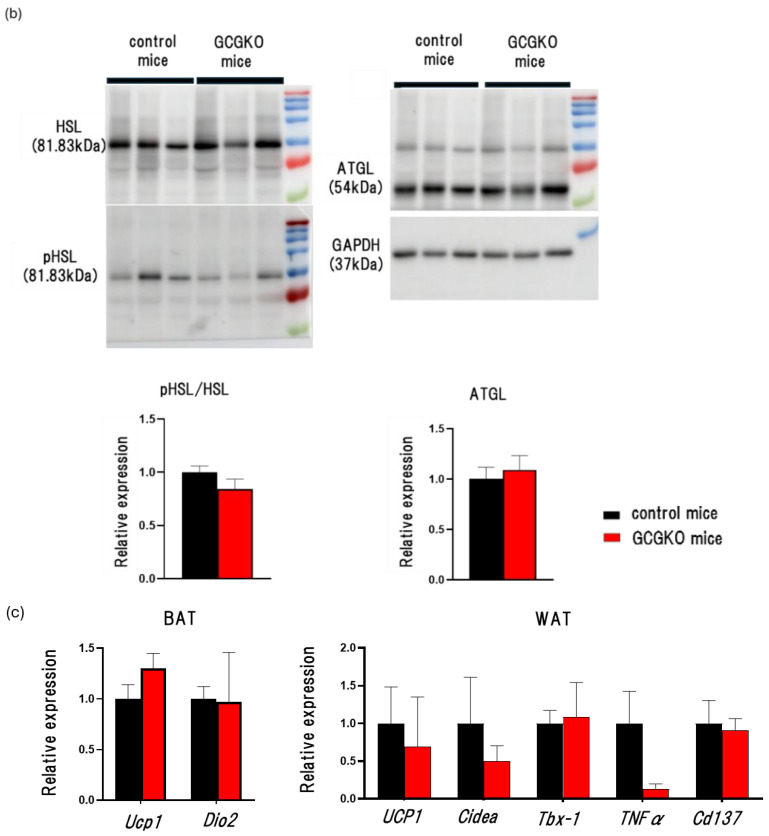
Phosphorylation of HSL in WAT and the expression levels of UCP1 mRNA in BAT and WAT did not differ between HFD-fed control mice and HFD-fed GCGKO mice. (**a**) Hematoxylin and eosin stain of WAT in NC-fed and HFD-fed control mice and NC-fed and HFD-fed GCGKO mice. (**b**) Quantification of HSL phosphorylation (pHSL) and ATGL of WAT in HFD-fed control mice and HFD-fed GCGKO mice. HFD-fed control mice (black bars; *n* = 6) and HFD-fed GCGKO mice (red bars; *n* = 6). (**c**) mRNA expression levels of the genes (Ucp1, Dio2) expressed in BAT and (Ucp1, Cidea, Tbx-1, Tnfα, Cd137) in the WAT of HFD-fed control mice (black bars; *n* = 8) and HFD-fed GCGKO mice (red bars; *n* = 6) after 1 week of intervention. Data are expressed as mean ± SEM. Statistical comparisons were performed using unpaired Student’s *t*-test for (**b**,**c**).

While WAT weight was increased in control mice on HFD, WAT weight in HFD-fed GCGKO mice was not increased compared to that in NC-fed GCGKO mice (Figure 1b). We therefore investigated the morphology and gene expression in adipose tissues. The size and amount of fat droplets in HFD-fed GCGKO mice were less than in HFD-fed control mice by hematoxylin and eosin staining of WAT (Figure 4a). No significant difference in the expression levels of *Ucp1* and *Dio2* (deiodinase iodothyronine type II) mRNA in BAT was observed between HFD-fed control mice and HFD-fed GCGKO mice. While the white adipose cell size differed between HFD-fed control and GCGKO mice, no significant differences were observed in the expression levels of *Ucp1*, *Cidea* (cell death-inducing DNA fragmentation factor alpha-like effector A), *Tbx-1* (T-box transcription factor 1), *Tnfα* (Tumor Necrosis Factor) and *Cd137* (Cluster of differentiation 137) mRNA in WAT (Figure 4b). These results differ from the previous report that long-term HFD feeding increases the expression levels of *Ucp1* mRNA in BAT and WAT and induces the expression of *Tbx-1* and *Cd137* (molecular markers of brown-like adipocytes) mRNA in the WAT of GCGKO mice [27]. Quantification of the ratio of HSL phosphorylation (pHSL) to HSL and ATGL of WAT did not differ between the two groups (Figure 4c), indicating that the enhancement of lipolysis did not contribute to the resistance to fat accumulation in the WAT of HFD-fed GCGKO mice.

### 3.5. Fat Absorption from the Intestinal Tract Was Decreased in HFD-Fed GCGKO Mice Compared to That in HFD-Fed Control Mice

**Figure 5:**
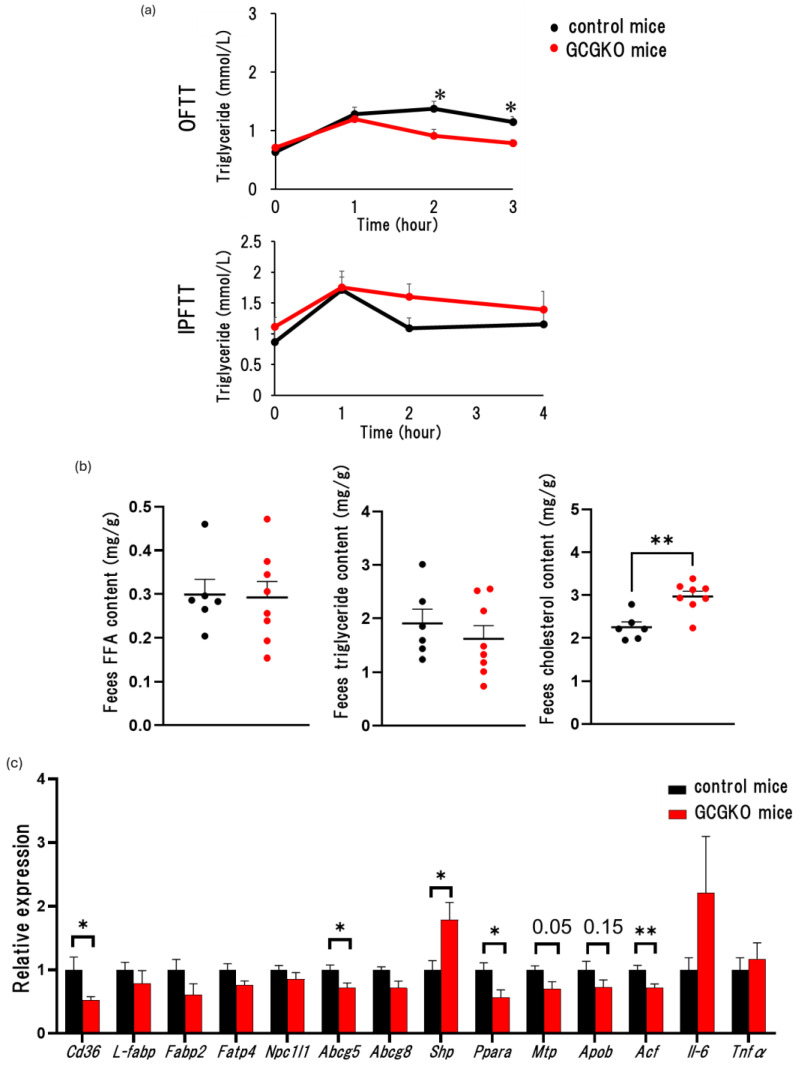

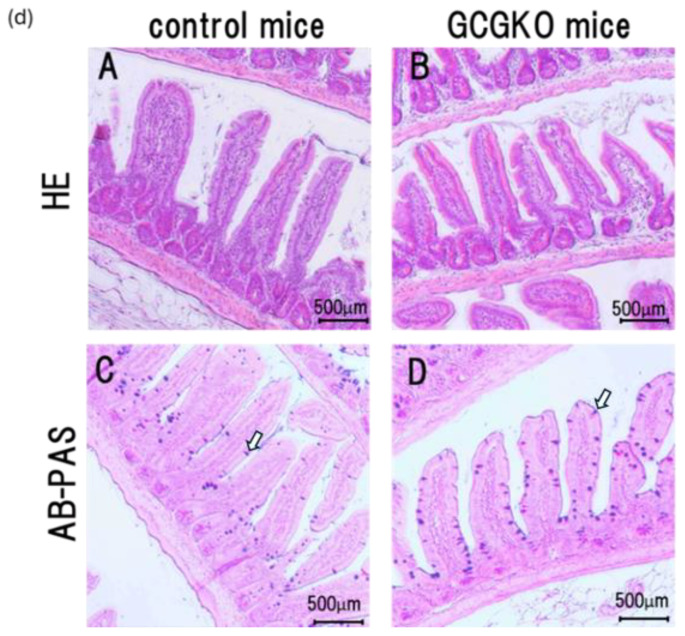
Fat absorption from the intestinal tract in HFD-fed GCGKO mice and HFD-fed control mice. (**a**) Time course of plasma triglyceride levels in oral fat tolerance test (OFTT) and intraperitoneal fat tolerance test (IPFTT) in control and GCGKO mice. Plasma triglyceride levels at 0, 1, 2, and 3 h in OFTT control mice (black line; *n* = 8), GCGKO mice (red line; *n* = 8), and at 0, 1, 2, and 4 h in IPFTT in control mice (black line; *n* = 9) and GCGKO mice (red line; *n* = 9). (**b**) Fecal FFA, triglycerides, and cholesterol content in HFD-fed control mice (black dots; *n* = 6) and HFD-fed GCGKO mice (red dots; *n* = 8). (**c**) mRNA expression levels of genes (*Cd36*, *L-fabp*, *Fabp2*, *Fatp4*, *Npc1l1*, *Abcg5*, *Abcg8*, *Shp*, *Pparα*, *Mtp*, *Apob*, *Acf*, *Il-6*, *Tnfα*) in HFD-fed control mice (black bars; *n* = 8) and HFD-fed GCGKO mice (red bars; *n* = 8) in the intestinal duodenum after 1 week intervention. (* *p* < 0.05, ** *p* < 0.01). (**d**) Representative photomicrographs of hematoxylin and eosin (HE) and alcian blue, periodic acid-Schiff (AB-PAS) staining in sections of the small intestine of HFD-fed control mice and GCGKO mice. Goblet cell production (arrows) could be observed at every segment. Data are expressed as mean ± SEM. Statistical comparisons were performed using an unpaired Student’s *t*-test.

We then evaluated fat absorption by the gastrointestinal tract in GCGKO and control mice. Plasma triglyceride levels were lower in GCGKO mice compared with those in control mice during the oral fat tolerance test (OFTT). On the other hand, plasma triglyceride levels did not differ between control mice and GCGKO mice during the intraperitoneal fat tolerance test (IPFFT) (Figure 5a). Fecal cholesterol content in HFD-fed GCGKO mice was significantly increased compared to that in HFD-fed control mice (Figure 5b). These results indicate that fat absorption is decreased in GCGKO mice. We then analyzed gene expression levels of mRNA in the duodenum of HFD-fed mice. The expression levels of Pparα, Cd36, Abcg5 (ATP binding cassette subfamily G member 5), and Acf (APOBEC1 complementation factor) were significantly lower in HFD-fed GCGKO mice compared with those in HFD-fed control mice. The expression levels of Mtp (microsomal triglyceride transfer protein) and Apob (apolipoprotein B) tended to be decreased in HFD-fed GCGKO mice compared with those in HFD-fed control mice. The expression levels of Shp (SH2-containing protein tyrosine phosphatase) were significantly higher in HFD-fed GCGKO mice compared with those in HFD-fed control mice (Figure 5c). There was no evident change in the morphology of the small intestine, including goblet cells in control and GCGKO mice fed HFD (Figure 5d).

### 3.6. The Gut Microbiomes Differed between HFD-Fed Control Mice and HFD-Fed GCGKO Mice

It has been reported that gut microbiota exerts metabolic effects [37] and that pancreatic-endocrine hormones such as insulin, GLP-1, and GLP-2 have a role in regulating the intestinal microbiome [38,39,40]. We therefore investigated for differences in the composition of the intestinal microbiome between the two groups.

**Figure 6:**
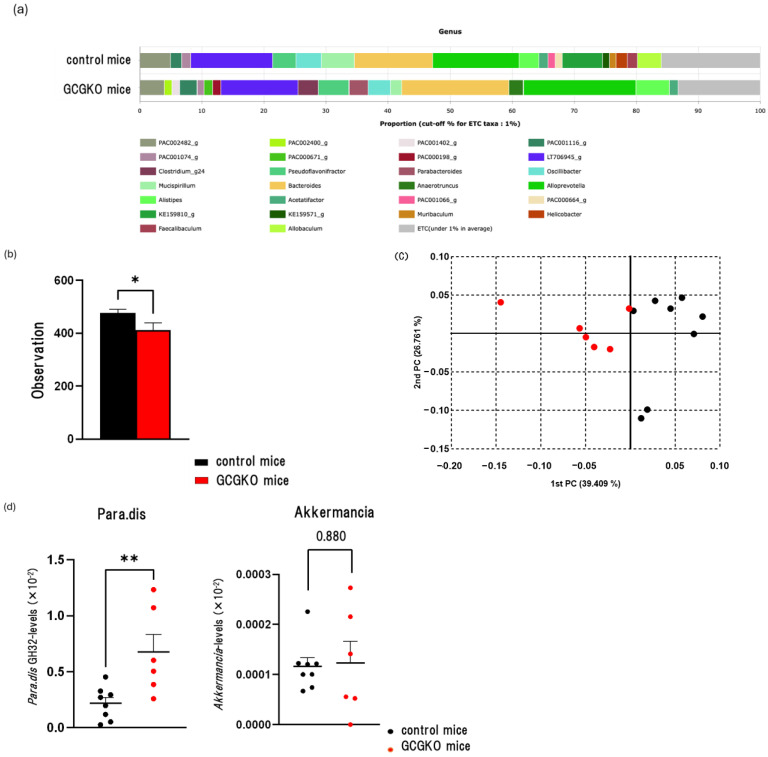
Gut microbiome in HFD-fed control mice and HFD-fed GCGKO mice. (**a**) Relative abundance of the taxonomic groups at the genus level in HFD-fed control mice and HFD-fed GCGKO mice. (**b**) Differences in alpha diversity indicated by phylogenetic diversity indices. (**c**) Principal coordinate analysis of beta diversity calculated by Jenson–Shannon divergence (*p* < 0.001). (**d**) Quantitative PCR (qPCR) Analysis of gene expression levels of *Parabacteroides distasonis* GH32 gene and the *Akkermansia muciniphila* Amuc_1434* gene in HFD-fed control (black dots; *n* = 8) and HFD-fed GCGKO mice (red dots; *n* = 6) after 1 week intervention. (* *p* < 0.05, ** *p* < 0.01). Data are expressed as mean ± SEM. Statistical comparisons were performed using unpaired Student’s *t*-test for (**b**,**d**).

An average of 28,903 ± 506 reads for the HFD-fed GCGKO mice group and 27,382 ± 865 reads for the HFD-fed control mice group were obtained. The datasets for NGS were deposited in the NCBI Sequence Read Archive under the accession number PRJNA1106176. In both groups, *Alloprevotella* was most abundant (18.14% and 13.93%, respectively) (Figure 6a).

Microbiome diversity indices were compared between the two groups. No significant differences in alpha diversity were observed, as indicated by the Chao1 (*p* = 0.12) and Shannon indices (*p* = 0.24). However, phylogenetic diversity indices were significantly lower in the HFD-fed GCGKO mice group compared to those in the HFD-fed control group (Figure 6b). The principal coordinate analysis (PCoA) plots for the groups showed distinct separation (Figure 6c), while PERMANOVA analysis of beta diversity revealed a significant difference in the gut microbiomes between the two groups (*p* < 0.001).

The LEfSe algorithm was used to identify potential genus-level biomarkers for differentiating the HFD-fed control mice group from the HFD-fed GCGKO mice group (Table 2). In the HFD-fed GCGKO mice group, the genera *Anaerotruncus* and *Parabacteroides* showed significantly higher relative abundances, as indicated by elevated LDA effect scores, compared to the HFD-fed control mice group. In contrast, the genera *Muribaculum* and *Lactobacillus* exhibited significantly lower relative abundances than in the HFD-fed control mice group.

We then performed qPCR on DNA samples to compare the abundance of *P. distasonis* and *A. muciniphila* between the two groups. The levels of the *P. distasonis* GH32 gene were significantly higher in the HFD-fed GCGKO group than in the HFD-fed control group (Figure 6d). On the other hand, the levels of the *A. muciniphila* Amuc_1434* gene showed no significant difference between these groups (Figure 6d).

## 4. Discussion

In the present study, we analyzed the role of PGDPs, including Gcg, GLP-1, and GLP-2, on lipid metabolism in mice fed HFD for 7 days using GCGKO mice. HFD-fed GCGKO mice showed lower white adipose tissue weight, lower FFA concentration in plasma, and lower hepatic content of FFA and triglycerides than HFD-fed control mice. We show that decreased lipid absorption from the intestinal tract is a major factor in these differences, despite the finding of decreased β-oxidation in the liver in HFD-fed GCGKO mice.

Under a fat-enriched diet, excessive accumulation of triglyceride in the liver is caused by multiple mechanisms [41]. HFD feeding increases the expression levels of *Cd36* mRNA in the liver, which enhances hepatic fat accumulation [42,43,44]. On the other hand, HFD feeding also increases the expression levels of *Pparα* and *Cpt1a*, which stimulate β-oxidation and production of ketone bodies and prevent excess fat accumulation [45]. Liver-specific PPARα-deficient mice fed NC or HFD show aggravated hepatic fat accumulation accompanied by decreased expression of genes involved in β-oxidation such as *Fgf21*, *Cpt1a,* and *Acox1* [46,47].

The ratio of insulin to glucagon is tightly linked to the regulation of metabolism in the liver. Insulin and Gcg act antagonistically not only in glucose metabolism but also in lipid metabolism [45]. Insulin inhibits gluconeogenesis and glycogenolysis, promotes lipogenesis, and suppresses β-oxidation, whereas Gcg stimulates gluconeogenesis and glycogenolysis, suppresses lipid synthesis, and promotes β-oxidation in the liver. Recently, data on the effects of glucagon agonism and antagonism on hepatic lipid metabolism-related genes have been rapidly accumulating [48,49,50]. The genes involved in fatty acid synthesis such as *Fas* (fatty acid synthase)*, Scd1, and Elovl6* (ELOVL fatty acid elongase 6) are increased in GcgRKO mice and mice treated with GcgR antibody for 8 weeks [48,49]. However, these two models of glucagon deficiency can exhibit differential data; the genes involved in fatty acid oxidation have been reported to be decreased and increased in GcgRKO mice and mice treated with GcgR antibody for 8 weeks [48,49]. As for glucagon agonism, exogenous glucagon administration has been shown to induce gluconeogenesis by stimulating ATGL-dependent lipolysis in the liver [50]. However, the role of endogenous glucagon on glucose and lipid metabolism in mice fed HFD has not been elucidated.

In the present study, we focused first on the liver, which plays a major role in the regulation of glucose and lipid metabolism. Blood glucose levels, liver glycogen contents, and the expression levels of *Pepck* (phosphoenolpyruvate carboxykinase) and *G6p* (Glucose-6-phosphate) did not differ between GCGKO and control mice fed HFD, indicating that Gcg plays a minor role in the regulation of glucose metabolism during HFD feeding for 7 days. On the other hand, the expression levels of genes involved in β-oxidation were decreased in GCGKO mice fed HFD compared with those in control mice fed HFD, suggesting that Gcg regulates β-oxidation in the liver under HFD-fed conditions. It has been reported that hepatic lipid accumulation is promoted due to impaired β-oxidation during HFD feeding in liver-specific PPARα-deficient mice [46,47]. Indeed, HFD-fed GCGKO mice displayed less fat accumulation in the liver than HFD-fed control mice, despite decreased expression of β-oxidation-related genes in the liver. However, the expression levels of genes involved in lipogenesis did not differ between GCGKO and control mice fed HFD. Thus, reduced triglyceride accumulation in GCGKO liver is likely due to decreases in FFA uptake in the liver, FFA release from adipose tissue, and/or lipid absorption from the gastrointestinal tract.

CD36 in the liver plays a major role in hepatic FFA uptake. CD36 overexpression in the liver increases FFA uptake and triglyceride synthesis [42]. In liver-specific CD36-deficient mice, triglyceride contents are decreased compared with those in control mice without alteration of the plasma FFA concentrations under HFD-feeding [44]. Thus, the reduced expression levels of CD36 in the liver in HFD-fed GCGKO mice compared to those in HFD-fed control mice may be partially involved in the lesser hepatic lipid accumulation. In addition, insulin increases CD36 expression levels in isolated hepatocytes and perfused livers [51], suggesting that the decreased insulin action caused by the lack of glucagon action [30] may have been involved in the decreased expression levels of CD36 in HFD-fed GCGKO mice.

The expression of glucagon receptors in adipose tissue is low compared to that in the liver [52]. Experiments on adipose tissue-specific GcgRKO mice indicated that the direct action of glucagon plays a minor role in lipolysis in white adipose tissue [53]. Because no significant difference in phosphorylation of HSL in white adipose tissue was observed between GCGKO and control mice, lipolysis in WAT is not likely to be enhanced in GCGKO mice. However, the metabolic status of the liver, including the intracellular amino acid concentration and glycogen content, has been reported to regulate lipid metabolism and lipolysis in WAT through neuronal liver/WAT communication [54,55]. Glucagon regulates this metabolic process and modulates the increase in fat mass in response to HFD feeding, as has been described in liver-specific GcgRKO mice [14]. Thus, glucagon might have mediated lipolysis in WAT indirectly through a neural pathway. We therefore performed hepatic vagotomy [56] in the GCGKO and control mice to ascertain whether a neural signal is involved in the differential increase in WAT weight under HFD in these mice. Hepatic vagotomy did not significantly affect WAT weight in either GCGKO or control mice (Figure S1), indicating that neural signals played a minor role in the regulation of WAT weight under the present experimental conditions.

On the other hand, lipid absorption from the intestinal tract in the present study revealed lower plasma triglyceride levels during olive oil loading in GCKKO mice, likely due to decreased lipid absorption and increased fecal cholesterol contents during HFD feeding. These changes could well account for the decreased fat accumulation and reduced plasma FFA concentrations observed in this study in HFD-fed GCGKO mice. While GcgRKO mice have been shown to exhibit enhanced lipid absorption during olive oil loading tests [49], the models have distinctly different plasma GLP-1 and GLP-2 levels, being extremely high in GcgRKO [22] and absent in GCGKO mice. In fact, GLP-1 reduces and GLP-2 increases postprandial lipid absorption from the intestinal tract [24,25,26]. In addition, GLP-2-deficient mice tended to show less numerical increase in plasma triglyceride levels after olive oil loading compared to control mice, although the difference did not reach statistical significance [57]. Nevertheless, blockade of GLP-2 action promotes hepatic triglyceride accumulation under both NC feeding [57] and HFD feeding [58] in mice due to markedly decreased insulin sensitivity [59]. Thus, the decreased triglyceride accumulation in the liver of GCGKO mice fed HFD is most likely caused by impaired lipid absorption due to an absence of GLP-2 action and enhanced insulin sensitivity by the blockade of Gcg action [28,29].

It has been reported recently that intestine-specific PPARα-deficient mice fed HFD showed reduced hepatic triglyceride accumulation in the liver and resistance to obesity. In these mice, *Cd36* mRNA is decreased in the intestinal tract and liver, which is thought to account for the reduced lipid absorption from the intestinal tract and reduced uptake of FFA in the liver [60]. Interestingly, in the present study, GCGKO mice both had lower expression levels of *Cd36* and *Pparα* mRNA in the intestine and liver, although the difference did not reach statistical significance in the liver. Moreover, exogenous administration of GLP-2 has been shown to increase the expression levels of intestinal glycosylated CD36 in hamsters, and the increase in postprandial TRL-apoB48 mass induced by GLP-2 is eliminated in CD36-deficient mice [26]. These data suggest that GLP-2 increases lipid absorption from the intestinal tract via upregulation of CD36 [26]. Thus, the absence of GLP-2 in the GCGKO mice may well have resulted in reduced expression of CD36 and impaired lipid absorption. In addition, PPARα expression is regulated by Gcg in the liver under fasting conditions [45,61], suggesting that the absence of not only GLP-2 but also Gcg may have contributed to the lower expression of *Pparα* mRNA in the HFD-fed GCGKO mice in the present study.

It should also be noted that pancreatic endocrine hormones such as insulin, GLP-1, and GLP-2 are involved in the regulation of the intestinal microbiome [38,39,40]. Isoxanthohumol (IX) has recently been reported to increase *Akkermansia muciniphila* in mice and to decrease fat absorption from the intestinal tract. In these experiments, IX administration reduced expression levels of *PPAR α* and *CD36* mRNA in the intestine concomitant with increased *Parabacteroides* and decreased *Lactobacillus*, and the mice showed resistance to obesity by HFD [62]. In addition, at the genus level, *Blautia*, *Anaerotruncus*, *Parabacteroides,* and *Alistipes* were shown to be negatively correlated with BMI in Chinese male college students [63]. In the present study, we found that *Parabacteroides* was significantly increased and *Lactobacillus* was significantly decreased in HFD-fed GCGKO mice compared to those in HFD-fed control mice, while *Akkermansia* did not differ in the two groups.

An important limitation of the present study is our inability to distinguish the contributions of Gcg, GLP-1, and GLP-2 in lipid metabolism in GCGKO mice, as they all lack PDGPs. Because of the short biological half-life of PDGPs, substitution experiments are challenging. Another limitation is the lack of observation of the effects of HFD on each organ over time. However, as differences in body weight may affect fat accumulation in the liver in HFD-fed mice [18], we chose in this study to conduct the analyses one week after HFD feeding before differences in body weight by HFD feeding manifested. While resistance to adipose tissue weight gain in GCGKO mice after 15 weeks of HFD has been reported [27], it is caused by enhanced energy expenditure rather than by decreased lipid absorption from the intestine tract, as it is after 1 week of HFD. Therefore, the mechanism of hepatic fat accumulation during HFD feeding varies depending on the duration of HFD feeding. Yet another limitation is that we cannot describe the underlying mechanism of the liver weight gain seen in HFD-fed GCGKO mice after only one week of HFD feeding. We recently found that GCGKO mice have greater muscle mass than controls due to enhancement of the IGF-1–protein kinase B pathway [52]. The contribution of this signaling pathway in the short-term gain in liver weight in HFD-fed GCGKO mice will be investigated in a future study.

## 5. Conclusions

In the present study, we analyzed lipid metabolism in mice fed HFD in the presence and absence of proglucagon-derived peptides. GCGKO mice show less increase in adipose tissue weight, plasma FFA, and hepatic triglyceride and FFA content under HFD, almost certainly due primarily to decreased lipid absorption from the intestinal tract. Decreased expression of CD36 and PPARα may contribute to the reduced lipid absorption observed in GCGKO mice. Our findings suggest a therapeutic avenue for the prevention and amelioration of fatty liver disease.

## Figures and Tables

**Table 1:** Nucleotide sequence alignment of *Akkermansia muciniphila* Amuc_1434* gene homologs. The sequence shown above the alignment indicates the region selected for primer sequence design. CP042830.1: *Akkermansia muciniphila* strain DSM 22959, CP048438.1: *Akkermansia muciniphila* strain JCM 30893, CP084201.1 *Akkermansia muciniphila* strain NBRC 114322, CP010553.1: *Akkermansia muciniphila* strain H2, and CP025823.1: *Akkermansia muciniphila* strain EB-AMDK-7.

	CATYGGCTGTTATCCGCAGC
CP042830.1	TT G AA C G A CC GG TT C A G C T A T GG AA T C GG C A T C GG C T G TT A T CC G C A G C TT GGGG A TT A T CC TTT CC T G CCCC TT A T CC A G TTT AAA T GG
CP048438.1	TT G AA C G A CC GG TT C A G C T A T GG AA T C GG C A TT GG C T G TT A T CC G C A G C TT GGGG A TT A T CC TTT CC T G CCCC TT A T CC A G TTT AAA T GG
CP084201.1	TT G AA C G A CC GG TT C A G C T A T GG AA T C GG C A TT GG C T G TT A T CC G C A G C TT GGGG A TT A T CC TTT CC T G CCCC TT A T CC A G TTT AAA T GG
CP010553.1	TT G AA C G A CC GG TT C A G C T A T GG AA T C GG C A T C GG C T G TT A T CC G C A G C TT GGGG A TT A T CC TTT CC T G CCCC TT A T CC A G TTT AAA T GG
CP025823.1	TT G AA C G A CC GG TT C A G C T A T GG AA T C GG C A T C GG C T G TT A T CC G C A G C TT GGGG A TT A T CC TTT CC T G CCCC TT A T CC A G TTT AAA T GG
	A GG T G A G C G A T GGG TT G AA G
CP042830.1	G AA G C TT CCC G C AA TT GG A C G C TT C A G C T GG AA GGGG CCC G CC TTT CC T A T A T C AA C AA GG T G A G C G A T GGG TT G AA G T GGGG A CCC TT C
CP048438.1	G AA G C TT CCC G C AA TT GG A C G C TT C A G C T GG AA GGGG CCC G CC TTT CC T A T A T C AA C AA GG T G A G C G A T GGG TT G AA G T GGGG A CCC TT C
CP084201.1	G AA G C TT CCC G C AA TT GG A C G C TT C A G C T GG AA GGGG CCC G CC TTT CC T A T A T C AA C AA GG T G A G C G A T GGG TT G AA G T GGGG A CCC TT C
CP010553.1	G AA G C TT CCC G C AA TT GG A C G C TT C A G C T GG AA GGGG CCC G CC TTT CC T A T A T C AA C AA GG T G A G C G A T GGG TT G AA G T GGGG A CCC TT C
CP025823.1	G AA G C TT CCC G C AA TT GG A C G C TT C A G C T GG AA GGGG CCC G CC TTT CC T A T A T C AA C AA GG T G A G C G A T GGG TT G AA G T GGGG A CCC TT C

**Table 2:** Results of the linear discriminant analysis effect size analysis at the genus level for distinguishing HFD-fed GCGKO mice from HFD-fed control mice, with criteria of |LDA effect size| > 3.0 and *p*-value < 0.05.

Taxon Name	LDA Effect Size	*p*-Value	Taxonomic Relative Abundance	
			Ctrl	GCGKO
*Anaerotruncus*	4.0296	0.001	0.26952	2.31236
*Muribaculum*	3.51248	0.001	1.06293	0.33961
*Lactobacillus*	3.02518	0.001	0.21994	0.03216
*ASTB_g*	3.20119	0.006	0.40544	0.04149
*Parabacteroides*	4.12931	0.006	0.56143	3.07072
*Odoribacter*	3.16979	0.014	0.34725	0.07691
*PAC001066_g*	3.5805	0.014	1.14625	0.44854
*Faecalibaculum*	3.78907	0.019	1.62686	0.25278
*KE159600_g*	3.25465	0.020	0.42597	0.08407
*PAC000664_g*	3.64829	0.020	1.15243	0.29079
*Helicobacter*	3.81307	0.038	1.81157	0.40755

## Data Availability

The data used to support the findings of this study are available from Y.S. (Yusuke Seino) upon request.

## Supplementary Materials

The following supporting information can be downloaded at: https://www.mdpi.com/article/10.3390/nu16142270/s1. Table S1: Primers used for quantitative real-time PCR (qPCR); Figure S1: Weight correction for liver weight, inguinal, and epididymal WAT in HFD-fed control mice, HFD-fed GCGKO mice, HFD-fed control SHAM mice, HFD-fed GCGKO SHAM mice, HFD-fed control HVX mice and HFD-fed GCGKO HVX mice.
