# Supplementary material for: Impaired Fat Absorption from Intestinal Tract in High-Fat Diet Fed Male Mice Deficient in Proglucagon-Derived Peptides
Source: Nutrients. 2024 Jul 14;16(14):2270. doi: 10.3390/nu16142270 (PMC11280123; doi:10.3390/nu16142270)
Supplement: Supplementary file 1 [file nutrients-16-02270-s001.zip › nutrients-3089100-supplementary.pdf]

Table S1. Primers used for quantitative real-time PCR (qPCR)

Gene	Forward primers (5′–3′)	Reverse primers (5′–3′)
Pparα	CCTGAACATCGAGTGTCGAATAT	GTTCTTCTTCTGAATCTTGCAGCT
Cd36	AGATGACGTGGCAAAGAACAG	CCTTGGCTAGATAACGAACTCTG
L-fabp	ATGAACTTCTCCGGCAAGTACC	GGTCCTCGGGCAGACCTAT
Fatp2	CTTCGGGAACCACAGGTCTTC	CATAGCAAGGCCTGTCCCATAC
Fatp5	TTCGAAAGAACCAACCCTTCCT	GCGTCGTACATTCGCAACAA
Hsl	GCAAGATCAAAGCCTCAGCG	GCCATATTGTCTTCTGCGAGTGT
Atgl	GTCCTTCACCATCCGCTTGTT	CTCTTGGCCCTCATCACCAG
Cpt1a	GCACTGCAGCTCGCACATTACAA	CTCAGACAGTACCTCCTTCAGGAAA
Acox1	GCCCAACTGTGACTTCCATC	GGCATGTAACCCGTAGCACT
Scd1	CTTCAAGGGCAGTTCTGAGG	CAATGGTTTTCATGGCAGTG
Fas	TTGCTGGCACTACAGAATGC	AACAGCCTCAGAGCGACAAT
Dgat1	TCCGTCCAGGGTGGTAGTG	TGAACAAAGAATCTTGCAGACGA
Dgat2	CCTTCCTGGTGCTAGGAGTG	CCAGTCAAATGCCAGCCA
Elovl6	GAAAAGCAGTTCAACGAGAACG	AGATGCCGACCACCAAAGATA
Srebp1c	GGAGCCATGGATTGCACATT	GAAGTCACTGTCTTGGTTGTTG
Chrebp	GATGGTGCGAACAGCTCTTCT	CTGGGCTGTGTCATGGTGAA
Acaca	GCCTCTTCCTGACAAACGAG	TGACTGCCGAAACATCTCTG
Pepck	GTGTTTGTAGGAGCAGCCATGAG	TAGCCGAAGAAGGGTCGCAT
G6p	CGGATCTACCTTGCTGCTCA	AACAAGAAGATGGTGATGAGACAAT
Ucp1	GGGCCCTTGTAAACAACAAA	GTCGGTCCTTCCTTGGTGTA
Dio2	GCTTCCTCCTAGATGCCTAC	TGGCTGAACCAAAGTTGACC
Cidea	TGCTCTTCTGTATCGCCCAGT	GCCGTGTTAAGGAATCTGCTG
Tbx-1	GGCAGGCAGACGAATGTTC	TTGTCATCTACGGGCACAAAG
Tnfα	CTGAACTTCGGGGTGATCGG	GGCTTGTCACTCGAATTTTGAGA

Cd137	CGTGCAGAACTCCTGTGATAAC	GTCCACCTATGCTGGAGAAGG
Fabp2	GTGGAAAGTAGACCGGAACGA	CCATCCTGTGTGATTGTCAGTT
Fatp4	ATCAACACCAACCTTAGGCG	AACCCTTGTCTGGGTGACTG
Npc1l1	TGTTTGGTATGGAGAGTGTGGA	GTCACAGCAGAGACTGACATTG
Abcg5	CCAGATTATGTGCATCTTAGGCA	CTGCTCAGAAAAACGTCGCT
Abcg8	CTGTGGAATGGGACTGTACTTC	GTTGGACTGACCACTGTAGGT
Shp	CTGGGAAGAAACAGGAACAAGA	GGCTCAGAAGTCATACAGAATA
Mtp	CATGTCAGCCATCCTGTTTG	CTCGCGATACCACAGACTGA
Apob	TGGGATTCCATCTGCCATCTCGAG	GTAGAGATCCATCACAGGACAATG
Acf	AGCCAGAATCCTGCAATCC	AGCATACCTCTTCGCTTCATCC
Il-6	CTTCACAAGTCGGAGGCTTA	TGCAAGTGCATCATCGTTGT
β-actin	CATCCGTAAAGACCTCTATGCCAAC	ATGGAGCCACCGATCCACA

Table S1 Nishida K et al

Figure S1 Materials and Method

Hepatic-selective vagotomy

Selective hepatic vagotomy and sham operation were performed as previously described [1]. After the surgery, sham-operated and hepatic-vagotomized(HVX) mice were maintained under NC diet for 5 days and then exposed to HFD for 7 days. The mice were sacrificed after feeding with isoflurane (MSD Animal Health; Tokyo, Japan). Liver and white adipose tissue (WAT) were collected and weighed.

Reference

1. Iwasaki, Y.; SendoM.; DezakiK.; Hira T.; Sato, T.; Nakata, M.; Goswami, C.; Aoki, R.; Arai, T.; Kumari, P. et al. GLP-1 releaseand vagal afferent activation mediate the beneficial metabolic and chronotherapeutic effects of D-allulose. Nat Commun. 2018, 9, 113.

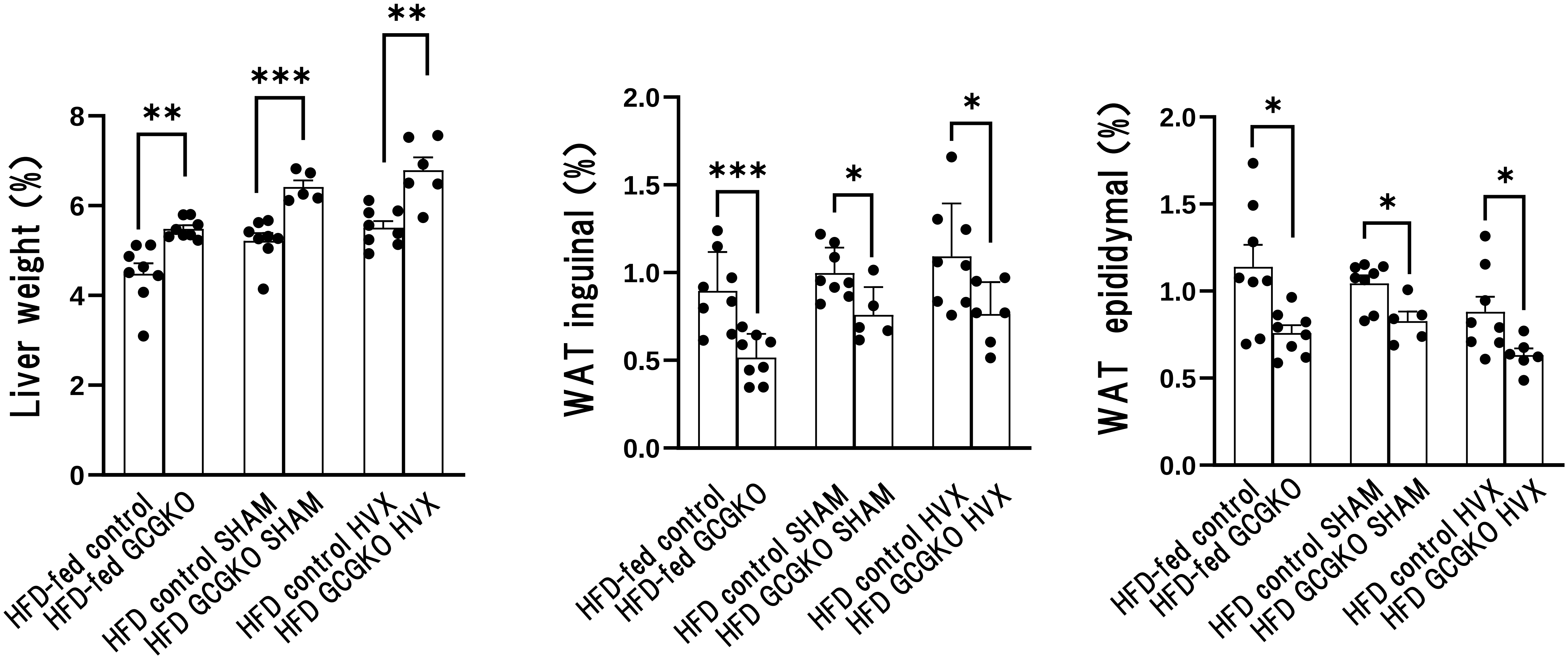

Supplemental Figure legend

Figure S1

Weight correction for liver weight, inguinal, and epididymal WAT in HFD-fed control mice (black dots; $n = 8$); HFD-fed GCGKO mice (black dots; $n = 8$); HFD-fed control SHAM mice (black dots; $n = 8$); HFD-fed GCGKO SHAM mice (black dots; $n = 5$); HFD-fed control HVX mice (black dots; $n = 8$); and HFD-fed GCGKO HVX mice (black dots; $n = 6$). Analysis was by t-test. (* $p < 0.05$, ** $p < 0.01$, *** $p < 0.001$). Data are expressed as mean ± SEM
